# Supplementary material for: Spring haul-out behavior of seals in the Bering and Chukchi Seas: implications for abundance estimation
Source: PeerJ. 2024 Oct 10;12:e18160. doi: 10.7717/peerj.18160 (PMC11471145; doi:10.7717/peerj.18160)
Supplement: Supplemental Information 1 [file peerj-12-18160-s001.pdf]

SUPPLEMENTAL MATERIAL

0.1 Additional Bio-logger Deployment Details

Table S1. The timing, location, and institutions responsible for the bio-logger deployments used in this study along with research permits and any associated publications.

Institution	Year Deployed	Location	Publication(s)	Permits	No. Seals	Sex	Age Class
**Bearded seal**							
ADFG	2005	Kotzebue Sound	Cameron et al 2018	358-1585	6	M, F	subadult
ADFG	2006	Kotzebue Sound	Cameron et al 2018	358-1585	2	F, M	subadult
ADFG	2009	Kotzebue Sound	Breed et al 2018	358-1787	4	F, M	subadult
ADFG	2014	Norton Sound, Koyuk River	Olnes et al 2020	15324	2	M	subadult
ADFG	2014	Norton Sound, Nome	Olnes et al 2020	15324	1	M	subadult
ADFG	2015	Norton Sound, St. Michael	Olnes et al 2020	15324	1	M	subadult
ADFG	2016	Elson Lagoon, Utqiagvik	Olnes et al 2020	15324	1	F	subadult
ADFG	2016	Norton Sound, Koyuk River	Olnes et al 2020	15324	2	F, M	subadult
ADFG	2016	Norton Sound, Nome	Olnes et al 2020	15324	1	M	subadult
ADFG	2016	Norton Sound, St. Michael	Olnes et al 2020	15324	2	M, F	subadult
ADFG	2017	Colville River, Nuiqsut	Olnes et al 2020	15324	1	F	subadult

Institution	Year Deployed	Location	Publication(s)	Permits	No. Seals	Sex	Age Class
ADFG	2017	Norton Sound, Koyuk River	Olnes et al 2020	15324	1	F	subadult
ADFG	2017	Norton Sound, Nome	Olnes et al 2020	15324	1	F	subadult
ADFG	2019	Dease Inlet, Utqiagvik	Olnes et al 2021	20466	1	M	adult
NMFS	2005	Kotzebue Sound		358-1585	1	F	subadult
NMFS	2009	Kotzebue Sound	McClintock et al 2017	782-1765	2	M	subadult, adult
NMFS	2011	Kotzebue Sound	McClintock et al 2017	15126	3	F, M	subadult
NMFS	2012	Kotzebue Sound	McClintock et al 2017	15126	1	F	adult
NSB	2012	Elson Lagoon, Utqiagvik		15324	1	M	subadult
NSB	2019	Pittalugruaq Lake		20466	1	F	subadult
**Ribbon seal**							
NMFS	2005	Ozemoy Gulf, Russia		782-1765	9	F, M	young of year, adult, subadult
NMFS	2006	Bering Sea		782-1765	7	M, F	adult, young of year
NMFS	2007	Bering Sea		782-1765	28	M, F	subadult, adult, young of year
NMFS	2008	Bering Sea		782-1765	1	M	subadult
NMFS	2009	Bering Sea		782-1765	28	F, M	adult, subadult, young of year

Institution	Year Deployed	Location	Publication(s)	Permits	No. Seals	Sex	Age Class
NMFS	2010	Bering Sea		358-1787, 15126	17	M, F	young of year, adult, subadult
NMFS	2014	Bering Sea		15126	13	M, F	subadult, adult, young of year
NMFS	2016	Bering Sea		19309	7	M, F	subadult, adult
**Spotted seal**							
ADFG	2005	Kotzebue Sound	Von Duyke et al in prep	358-1585	3	F, M	subadult, adult
ADFG	2016	Dease Inlet, Utqiagvik	Von Duyke et al in prep	15324	4	M, F	adult
ADFG	2017	Colville River, Nuiqsut	Von Duyke et al in prep	15324	1	F	subadult
ADFG	2017	Scammon Bay	Von Duyke et al in prep	15324	3	F, M	subadult, adult
ADFG	2018	Dease Inlet, Utqiagvik	Von Duyke et al in prep	20466	1	F	subadult
ADFG	2018	Scammon Bay	Von Duyke et al in prep	20466	1	M	subadult
ADFG	2019	Dease Inlet, Utqiagvik	Von Duyke et al in prep	20466	6	M	adult, subadult
NMFS	2006	Bering Sea		782-1676	5	M, F	young of year, subadult
NMFS	2007	Bering Sea		782-1676	12	F, M	adult, young of year, subadult
NMFS	2009	Bering Sea		358-1787	23	F, M	adult, subadult, young of year

Institution	Year Deployed	Location	Publication(s)	Permits	No. Seals	Sex	Age Class
NMFS	2010	Bering Sea		358-1787, 15126	8	F, M	young of year, adult, subadult
NMFS	2014	Bering Sea		15126	5	M, F	young of year, adult
NMFS	2016	Bering Sea		19309	6	M, F	adult
NMFS	2018	Bering Sea		19309	5	F	adult
NPWC	2009	Kamchatka Peninsula		NA	3	F	adult
NSB	2012	Tiny Island	Von Duyke et al in prep	15324	1	F	adult
NSB	2014	Oarlock Island	Von Duyke et al in prep	15324	6	M, F	subadult, adult
NSB	2014	Seal Island	Von Duyke et al in prep	15324	1	M	subadult
NSB	2015	Oarlock Island	Von Duyke et al in prep	15324	6	M, F	subadult, adult
NSB	2016	Pittalugruaq Lake	Von Duyke et al in prep	15324	3	F	subadult
NSB	2017	Pittalugruaq Lake	Von Duyke et al in prep	15324	1	M	subadult

ADFG=Alaska Department of Fish and Game; **NSB**=North Slope Borough; **NMFS**=NOAA National Marine Fisheries Service; **NPWC**=North Pacific Wildlife Consortium

0.2 Supplemental Figures Showing Confidence Intervals Associated with Predictions

The following series of figures (S1, S2, and S3) show the seasonal variability in predicted haul-out probability and the associated 95% confidence intervals for bearded, ribbon, and spotted seals. The predictions shown are based on the same data used in 5, 7, and 9 but selected for three local solar hours (07:00, 12:00, and 17:00) so the confidence intervals can also be shown and comparisons can be made.

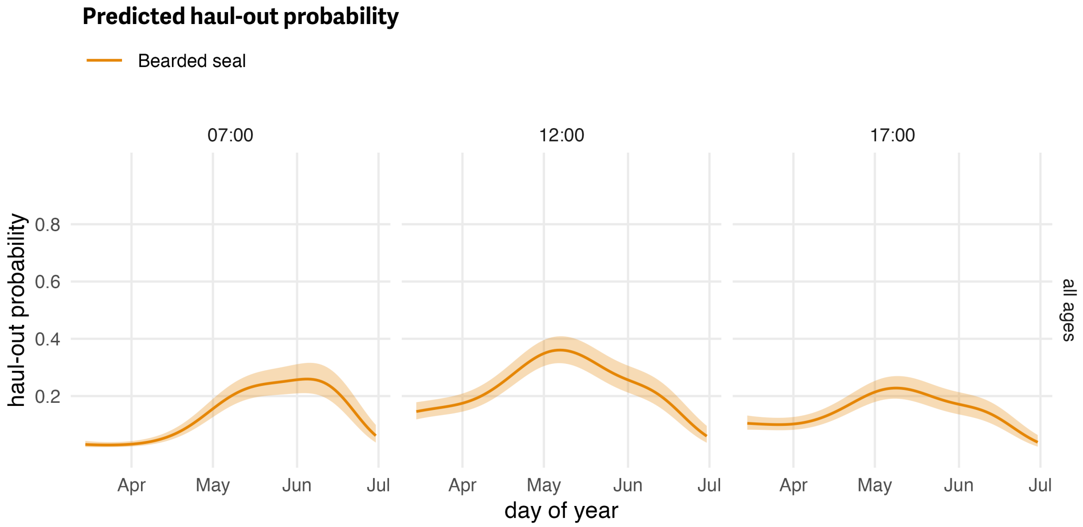

Figure S1. Seasonal variability in haul-out probability and the associated 95% confidence intervals (shaded area) for bearded seals.

Model predictions are shown for three local solar hours (07:00, 12:00, and 17:00). Weather covariate values in the prediction were based on a simple generalized additive model for each weather covariate with smooth terms for day-of-year and solar hour to account for anticipated variability within a day over the season. Age and sex classes are combined into a single ‘all ages’ category.

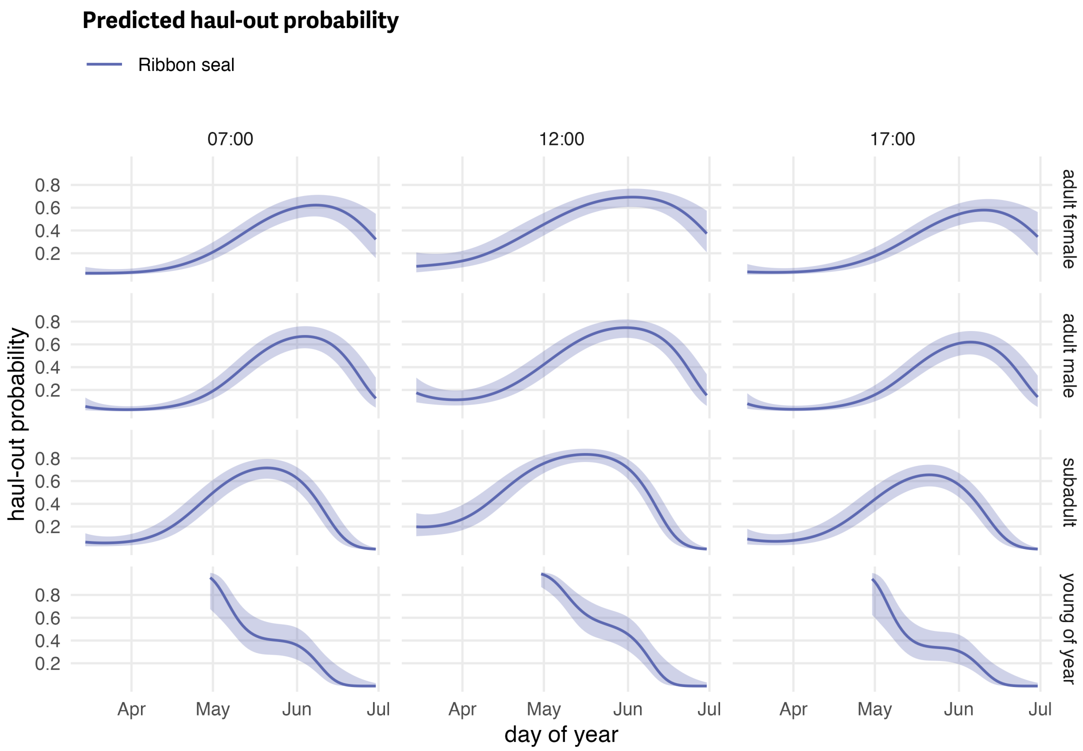

Figure S2. Seasonal variability in haul-out probability and the associated 95% confidence intervals (shaded area) for ribbon seals.

Model predictions are shown for three local solar hours (07:00, 12:00, and 17:00). Weather covariate values in the prediction were based on a simple generalized additive model for each weather covariate with smooth terms for day-of-year and solar hour to account for anticipated variability within a day over the season. Age and sex classes are separated to allow comparisons.

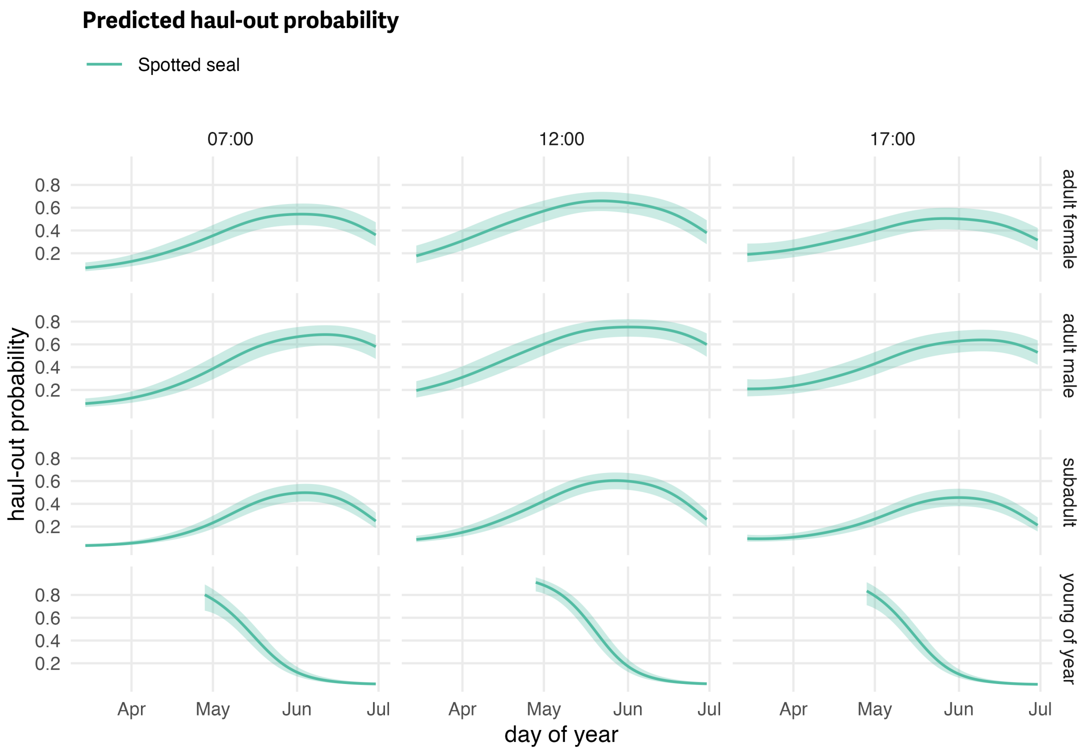

Figure S3. Seasonal variability in haul-out probability and the associated 95% confidence intervals (shaded area) for spotted seals.

Model predictions are shown for three local solar hours (07:00, 12:00, and 17:00). Weather covariate values in the prediction were based on a simple generalized additive model for each weather covariate with smooth terms for day-of-year and solar hour to account for anticipated variability within a day over the season. Age and sex classes are separated to allow comparisons.

0.3 Exploring Insolation (Solar Radiation) as a Model Covariate

0.3.1 Introduction

During the peer review process for this manuscript, Anthony Fischbach suggested the possibility of using predicted insolation (or solar radiation) values from the reanalysis model as a more direct and, potentially, more informative predictor of the daily haul-out cycle in seals compared to time of day. The notion being that seals are, likely, directly responding to changes in solar radiation throughout the day and not what time of day it is (i.e. seals don't have human watches). Additionally, given the energetic benefits of increased solar radiation it could be more informative as we would expect seals might have a higher haul-out probability on sunnier days and for there to be geographic variability in haul-out behavior associated with geographical differences in insolation. This approach has an additional benefit of being more parsimonious compared to our use of the Fourier series or other approaches to represent hour-of-day in the model (e.g. 24 factors for each hour).

Because of these reasons, we considered and explored this possibility for our model and the analysis presented in this manuscript. A key drawback to reliance on solar radiation, in our minds, is that we would lose insight regarding potential diel patterns – solar radiation does not differentiate between dusk or dawn. Bi-modal patterns have been previously observed in ringed seals and our results in this analysis show some indication of increased haul-out probability during dawn compared to dusk periods for bearded seals and some age and sex classes for ribbon and spotted seals. For other phocid species, increased haul-out probability before solar noon or after solar noon has been observed. Importantly, understanding these relationships between haul-out probability and hour-of-day can have important ramifications on aerial survey study design – a key focus of this paper.
Another hesitation we had was that solar radiation estimates from reanalysis models have not been previously used as a model covariate within a published study of pinniped haul-out behavior. Thus, for this analysis, we chose to keep our original approach and rely on the Fourier series to capture any hour-of-day effects.

That said, we think the idea of solar radiation as a model covariate in pinniped haul-out models is intriguing and worth further exploration. The current availability and increased accessibility to detailed climate reanalysis products that include solar radiation is exciting and we encourage future, more detailed exploration of this as a component in pinniped haul-out analysis. To provide some inspiration, we present some initial efforts and examples for comparison.

0.3.2 Methods

In this manuscript, we rely on the NARR reanalysis model as the source for our weather covariates. However, since our initiation of this analysis, the ERA5 reanalysis model (https://doi.org/10.24381/cds.adbb2d47) has become one of the go-to standards for global climate reanalysis and provides an increased temporal resolution to hourly (compared to the 3-hour resolution of NARR). The global coverage of ERA5 provides additional flexibility in that the area of interest is not limited to North America. The ERA5 model provides a number of solar radiation parameters and it was important to evaluate and understand each of these estimates in order to select the one that was likely most relevant to seals. Here, we used the 'surface short-wave (solar) radiation downwards' parameter. This parameter is described as *"the amount of solar radiation (also known as shortwave radiation) that reaches a horizontal plane at the surface of the Earth and comprises both direct and diffuse solar radiation. To a reasonably good approximation, this parameter is the model equivalent of what would be measured by a pyranometer (an instrument used for measuring solar radiation) at the surface"* (https://codes.ecmwf.int/grib/param-db/?id=169). Thus, this is the value

which most closely represents the amount of solar radiation likely felt by a seal hauled out of the water.

ERA5 data is available via the Copernicus climate data store API which can be queried with the CDS-API Python package (https://cds.climate.copernicus.eu/api-how-to). The R code provided here documents the download of the *surface_solar_radiation_downwards* parameter for our study area of interest and years of interest. The `reticulate` R package (https://CRAN.R-project.org/package=reticulate) allowed interaction with Python. Additionally, note, extra steps are required to download data on either side of the 180 anti-meridian.

```
library(tidyverse)
library(reticulate)
library(sf)
library(terra)

#import python CDS-API
cdsapi <- import('cdsapi')
#for this step there must exist the file .cdsapirc
server = cdsapi$Client() #start the connection

get_era5 <- function(y) {
  #we create the query
  query <- r_to_py(
    list(
      variable = "surface_solar_radiation_downwards",
      product_type = "reanalysis",
      area = "75/152/47/180", # North, West, South, East
      year = y,
      month = str_pad(2:7, 2, "left", "0"),
      day = str_pad(1:31, 2, "left", "0"),
      time = str_c(0:23, "00", sep = ":") %>% str_pad(5, "left", "0"),
      format = "netcdf"
    )
  )
  #query to get the ncdf
  server$retrieve("reanalysis-era5-single-levels",
                  query,
                  paste0("era5_ssrd_", y, "_left.nc"))

  query <- r_to_py(
    list(
      variable = "surface_solar_radiation_downwards",
      product_type = "reanalysis",
      area = "75/-180/47/-142", # North, West, South, East
      year = y,
      month = str_pad(2:7, 2, "left", "0"),
```

```
      day = str_pad(1:31, 2, "left", "0"),
      time = str_c(0:23, "00", sep = ":") %>% str_pad(5, "left", "0"),
      format = "netcdf"
    )
  )
  #query to get the ncdf
  server$retrieve("reanalysis-era5-single-levels",
                  query,
                  paste0("era5_ssrd_", y, "_right.nc"))

}

years <- as.character(2005:2021)
for(i in 1:length(years)) {
  get_era5(years[i])
}
```

To explore performance of our solar radiation parameter within a haul-out model we replaced the various Fourier series parameters in our model from the manuscript with the ERA5 *surface solar radiation downwards* (`era_ssrd_watts`) parameter. As with other reanalysis values (from NARR) in the manuscript, the `era-ssrd-watts` values are matched in time and space to the seal haul-out observation data; we use the full hourly temporal resolution from ERA5. The glmmLDTS framework used in the paper does not allow for model comparisons with AIC because of the reliance on pseudo-likelihood. The `bam()` function within the `mgcv` package provides a quick model fitting option that also allowed us to do some model comparison with AIC. This approach was sufficient for the general demonstration and exploration purposes here but future research should consider a range of model fitting frameworks and approaches that might be more appropriate.

The model specification below was used to specify an `mgcv::bam()` model that matched the formula used in the manuscript for ribbon seals. The `s(speno, bs = "re")` term is the smooth term for the random effect. All other predictors were the same.

```
m1_ribbon <- mgcv::bam(
  dry ~ age_sex + s(speno, bs = "re") +
    sin1 + cos1 + sin2 + cos2 + sin3 + cos3 +
    poly(day, 3, raw=TRUE) +
    sin1:poly(day, 3, raw=TRUE) +
    cos1:poly(day, 3, raw=TRUE) +
    sin2:poly(day, 3, raw=TRUE) +
    cos2:poly(day, 3, raw=TRUE) +
    sin3:poly(day, 3, raw=TRUE) +
    cos3:poly(day, 3, raw=TRUE) +
    wind*temp2m + pressure + precip +
    age_sex:poly(day, 4, raw=TRUE),
  data = ribbon_model_data,
  family = binomial,
```

```
  discrete = TRUE)
```

Note, the specification for *m1_ribbon* here does not include any AR1 structure for temporal autocorrelation. To include this, we needed to provide a value for ρ (or *rho*). We examined the autocorrelation within the model and used the lag-1 value for ρ .The value for lag-1 autocorrelation was 0.8082 which is rather high but not surprising. We then updated our model specification with a value for ρ as well as the `A1.start` argument which specifies (as either **TRUE** or **FALSE**) the start point of each block.

```
m2_ribbon <- mgcv::bam(
  dry ~ age_sex + s(speno, bs = "re") +
    sin1 + cos1 + sin2 + cos2 + sin3 + cos3 +
    poly(day, 3, raw=TRUE) +
    sin1:poly(day, 3, raw=TRUE) +
    cos1:poly(day, 3, raw=TRUE) +
    sin2:poly(day, 3, raw=TRUE) +
    cos2:poly(day, 3, raw=TRUE) +
    sin3:poly(day, 3, raw=TRUE) +
    cos3:poly(day, 3, raw=TRUE) +
    wind*temp2m + pressure + precip +
    age_sex:poly(day, 3, raw=TRUE),
  data = ribbon_model_data,
  family = binomial,
  AR.start = ar1_start,
  rho = lag1_ribbon,
  discrete = TRUE)
```

The model specification for exploring the use of solar radiation was specified similarly but without all of the Fourier series parameters and interactions.

```
m2_ssrd_ribbon <- mgcv::bam(
  dry ~ age_sex + s(speno, bs = "re") +
    era5_ssrd_watts +
    poly(day, 3, raw=TRUE) +
    era5_ssrd_watts:poly(day, 3, raw=TRUE) +
    wind*temp2m + pressure + precip +
    age_sex:poly(day, 3, raw=TRUE),
  data = ribbon_model_data,
  family = binomial,
  AR.start = ar1_start,
  rho = lag1_ribbon,
  discrete = TRUE)
```

The two models were compared with AIC to evaluate whether the reduction in degrees of freedom with fewer terms in the solar radiation model was matched with improved explanatory power in

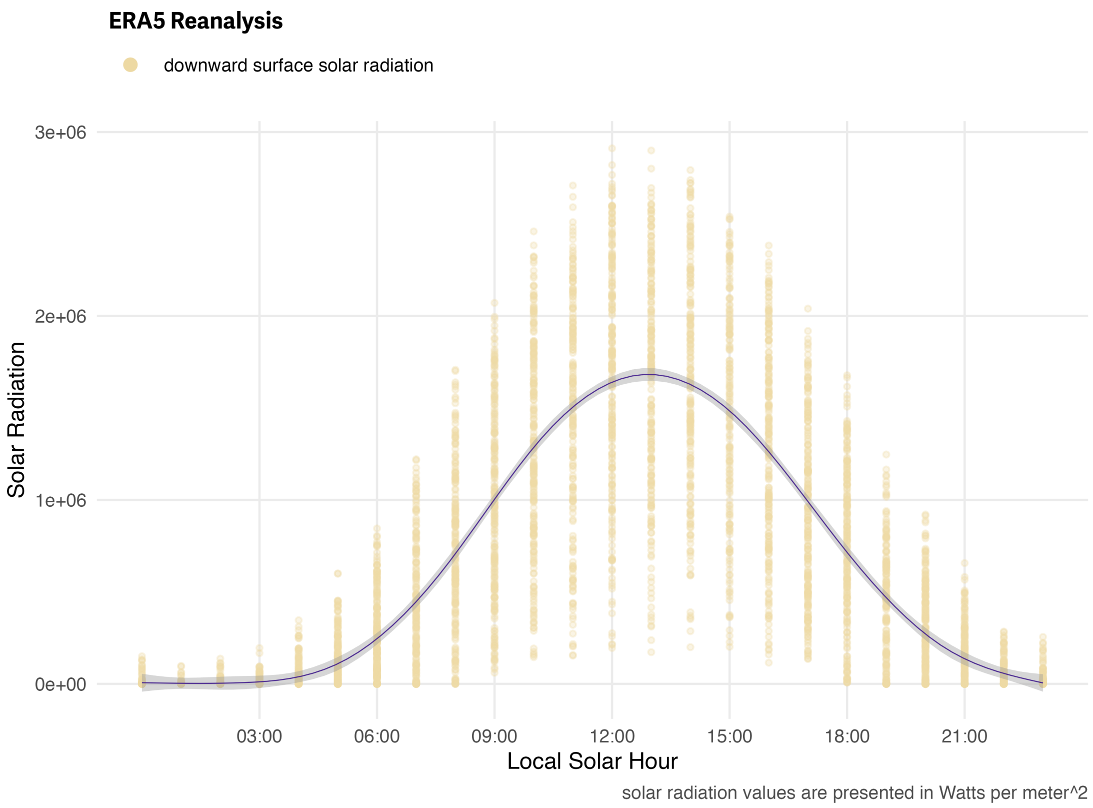

Figure S4. Diel Pattern of Solar Radiation Values from ERA5 Reanalysis. Downward surface solar radiation estimates from the ERA5 climate reanalysis for 5000 random points within the study area between 2005 and 2021. Solar radiation values are presented in Watts per square-meter and the smoothed line highlights the strong diel pattern.

the model fit. While the model and code specified above is for ribbon seals, the same approach was repeated for bearded and spotted seals.

A similar approach to that presented in this manuscript for prediction was employed with solar radiation values in lieu of hour of day. For prediction values, quantiles (5% increments) of the observed range of ERA5 solar radiation values were used with 100% representing the maximum observed solar radiation value. This allowed similar data visualizations and easier comparisons to those predictions in the manuscript that include hour of day.

0.3.3 Results

To evaluate whether the solar radiation parameter matched our expectations and compared well with hour of the day, we visualized the variability of the `era5_ssrd` values within our study area as they relate to hour of the day (S4). The unimodal distribution is centered around the middle of the solar day with peak solar radiation coinciding with 13:00 local solar. This suggests solar radiation could be an informative covariate for capturing unimodal diel patterns in haul-out behavior.

The bearded seal model matching the specification from the manuscript resulted in 126.13 degrees of freedom and an AIC value of -7428.929. The model with solar radiation resulted in 39.619 degrees of freedom and an AIC value of -6797.378. The ribbon seal model matching the specification from the manuscript resulted in 131.478 degrees of freedom and an AIC value of -16372.29. The model

with solar radiation resulted in 115.126 degrees of freedom and an AIC value of -16038.175. The spotted seal model matching the specification from the manuscript resulted in 125.506 degrees of freedom and an AIC value of -23584.373. The model with solar radiation resulted in 109.163 degrees of freedom and an AIC value of -23302.772. Despite the additional terms, the models with the Fourier series representation of hour of day resulted in a lower AIC value and were still preferred models for each of the species.

Predictions from the model fits and visualization of those predictions were produced for each species but, here, we only present visualizations from ribbon seals as an example (Figure S5 and Figure S6). Similar seasonal patterns previously observed were still apparent with subadults hauling out earlier in the season followed by adult males and, then, adult females. The observed relationship with hour of day and the centering of peak haul-out probability around solar noon was reflected in these predictions as a one-sided distribution with maximum solar radiation having the highest haul-out probability and minimal solar radiation the least. The seasonal distribution of haul-out probability along with 95% confidence intervals also provided comparable insights (see figures S2 and S6). That said, subtle differences in the shape and extent of confidence limits were present.

0.3.4 Discussion

Solar radiation has potential as an informative covariate in pinniped haul-out models that can be directly linked to seal physiology and expected behavioral changes. The ERA5's *surface solar radiation downwards* values aligned with hour of day and maximum values occurred at or just after local solar noon. This highlighted the informative potential for this approach. However, despite an overall reduction in the total number of parameters and degrees of freedom, AIC comparison still favored the models for each species that included hour of day as a Fourier series.

This analysis was not intended to be a full comparison – we simply want to demonstrate the potential and inspire further investigation – but, there are three possibilities that might explain the preference for hour of day. First, there are a broad range of solar radiation values represented for each hour of the day. Cloud cover, fog, and precipitation all reduce downward solar radiation at the surface and we might expect this to impact haul-out probability. However, the photoperiod and the timing of sunrise and sunset are not impacted by weather and seals may be responding to these signals more than the amount of solar radiation. Additionally, this study spans a range of physiological cycles and energetic needs and higher solar radiation may not be a consistent driving influence on seals. Increased energy from the sun may be important during molt but less so during pupping and breeding periods. Second, the timing and duration of haul-out behavior may also be influenced by diel patterns in weather (e.g. lower winds in the morning) or ecosystem dynamics (e.g. prey availability) that lead to a skewness in the distribution of haul-out behavior that wouldn't be reliably captured by solar radiation values. Third, this effort is only an initial effort to explore the use of solar radiation in pinniped haul-out models. A more in depth and rigorous exploration of this topic might discover an approach that results in a more parsimonious and preferred model formulation.

Again, we want to acknowledge Anthony Fischbach for the suggestion during the peer review process. We think this is an excellent example of the peer review process working to improve the quality of our manuscript and advance the scientific process. We hope others will take our example and expand on it within future analyses.

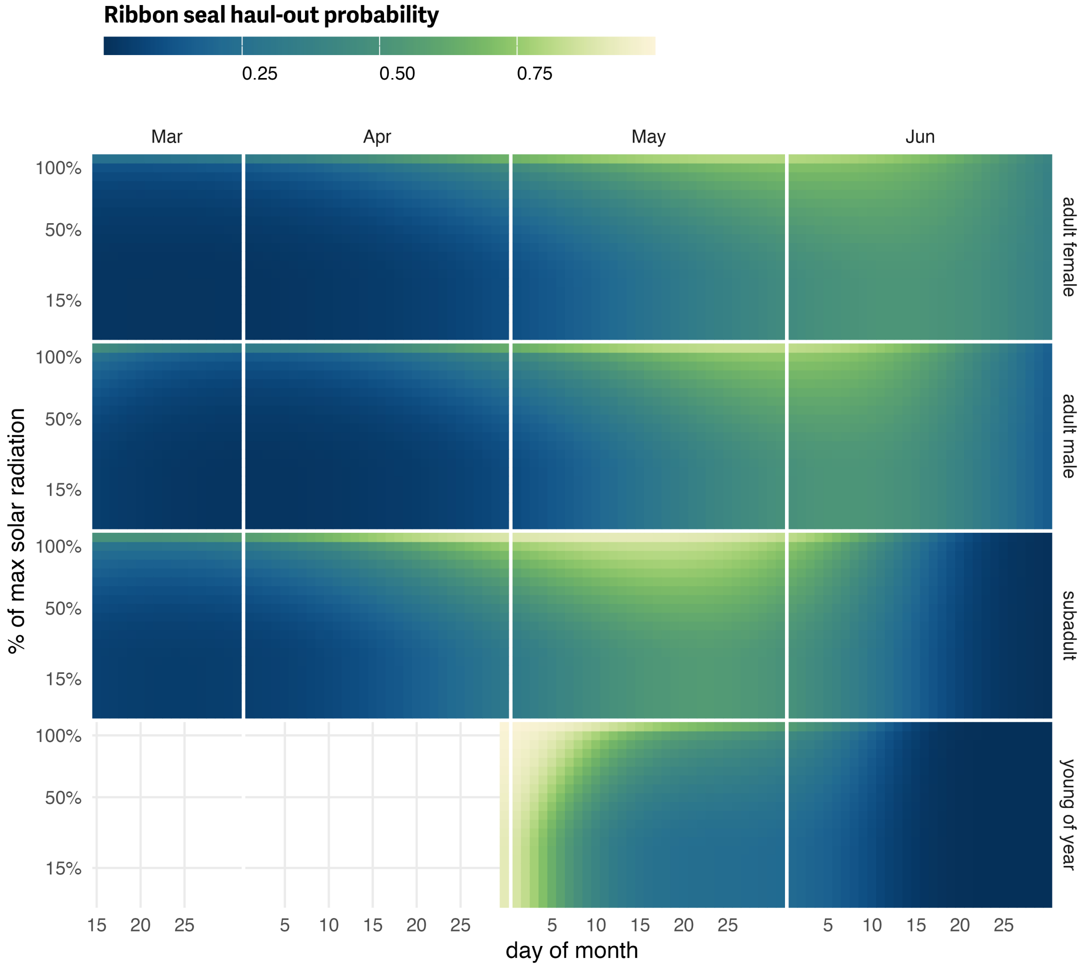

Figure S5. Solar radiation as a predictor of ribbon seal haul-out probability.
Predicted haul-out probability of ribbon seals from 15 March to 30 June for each age and sex class used in the model. In this model, solar radiation was used in lieu of hour of day. The apparent seasonal progression with subadults hauling out earlier in the season followed by adult males and, then, adult females is still notable although maybe not as clear. Predictions for young of the year still show their transition from newly weaned pups resting on the ice to more in-water activities. The overall pattern is in agreement with a one-sided view of Figure 7 where maximum solar radiation is equivalent to local solar noon.

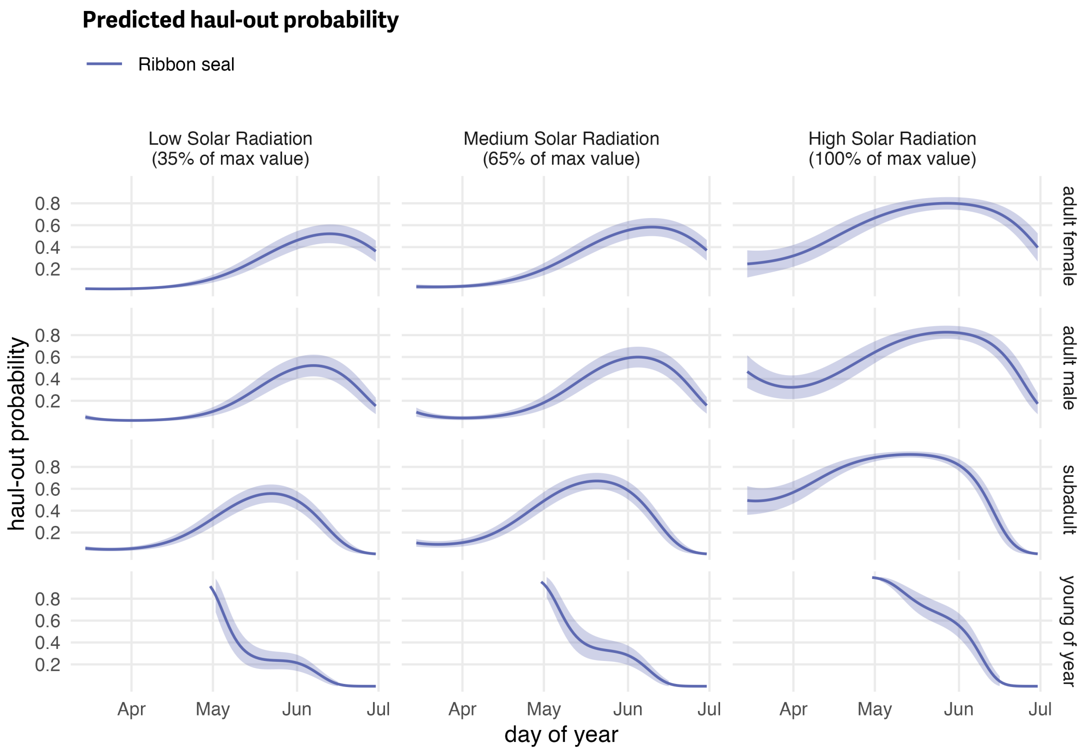

Figure S6. Solar radiation as a predictor of ribbon seal haul-out probability (with uncertainty).

Seasonal variability in haul-out probability and the associated 95% confidence intervals (shaded area) for ribbon seals. In this model predictions are shown for low, medium, and high values of solar radiation (as percentages of the maximum value observed) in lieu of local solar hour. There's general agreement in the overall seasonal patterns between the two approaches but sublte differences in shape and extent of the confidence limits were present (see Figure S2 for comparisons).
