# Supplementary figures and images for: Spring haul-out behavior of seals in the Bering and Chukchi Seas: implications for abundance estimation
Source: PeerJ. 2024 Oct 10;12:e18160. doi: 10.7717/peerj.18160 (PMC11471145; doi:10.7717/peerj.18160)

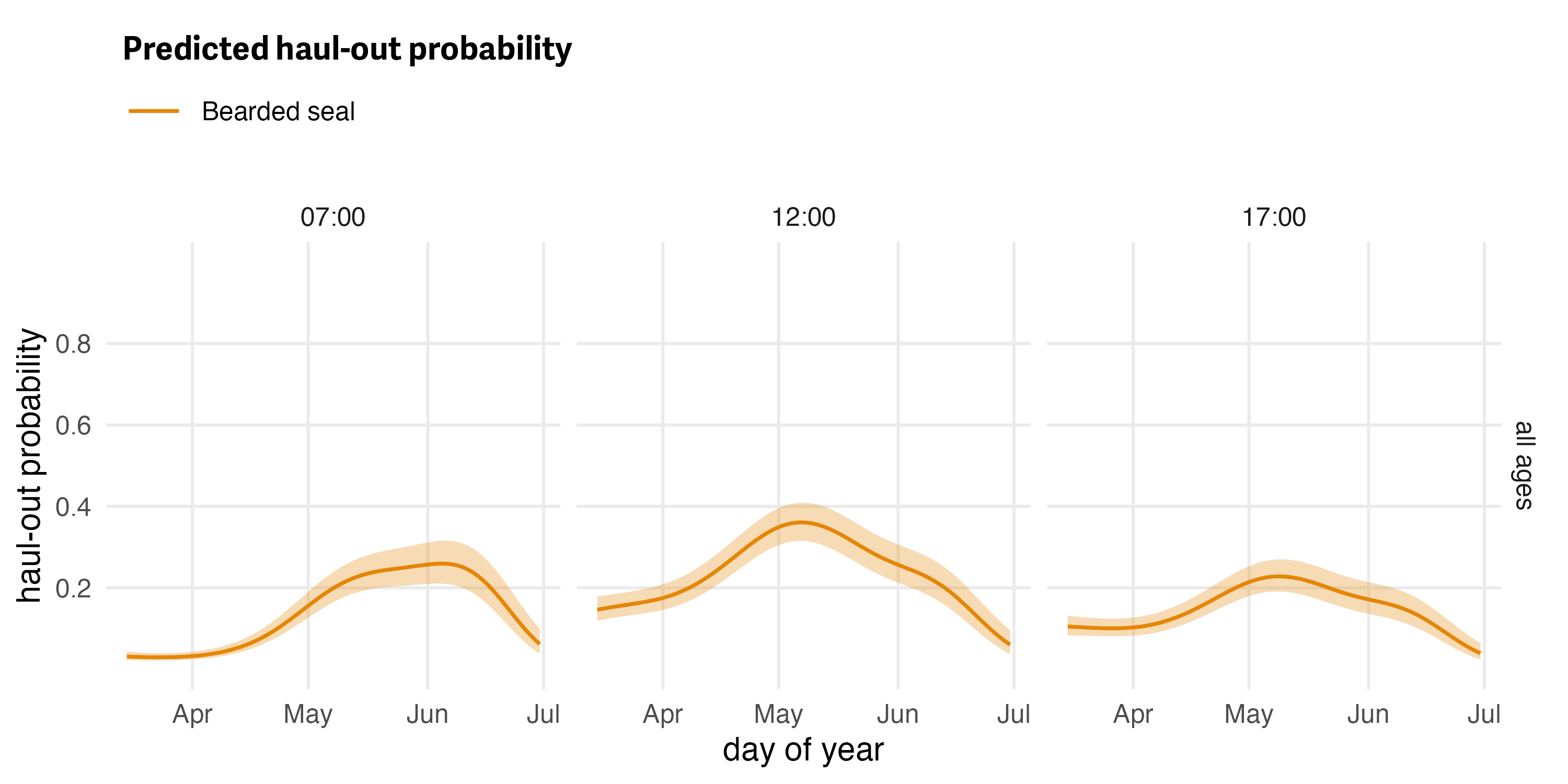

Supplement: Supplemental Information 2 [file peerj-12-18160-s002.png]

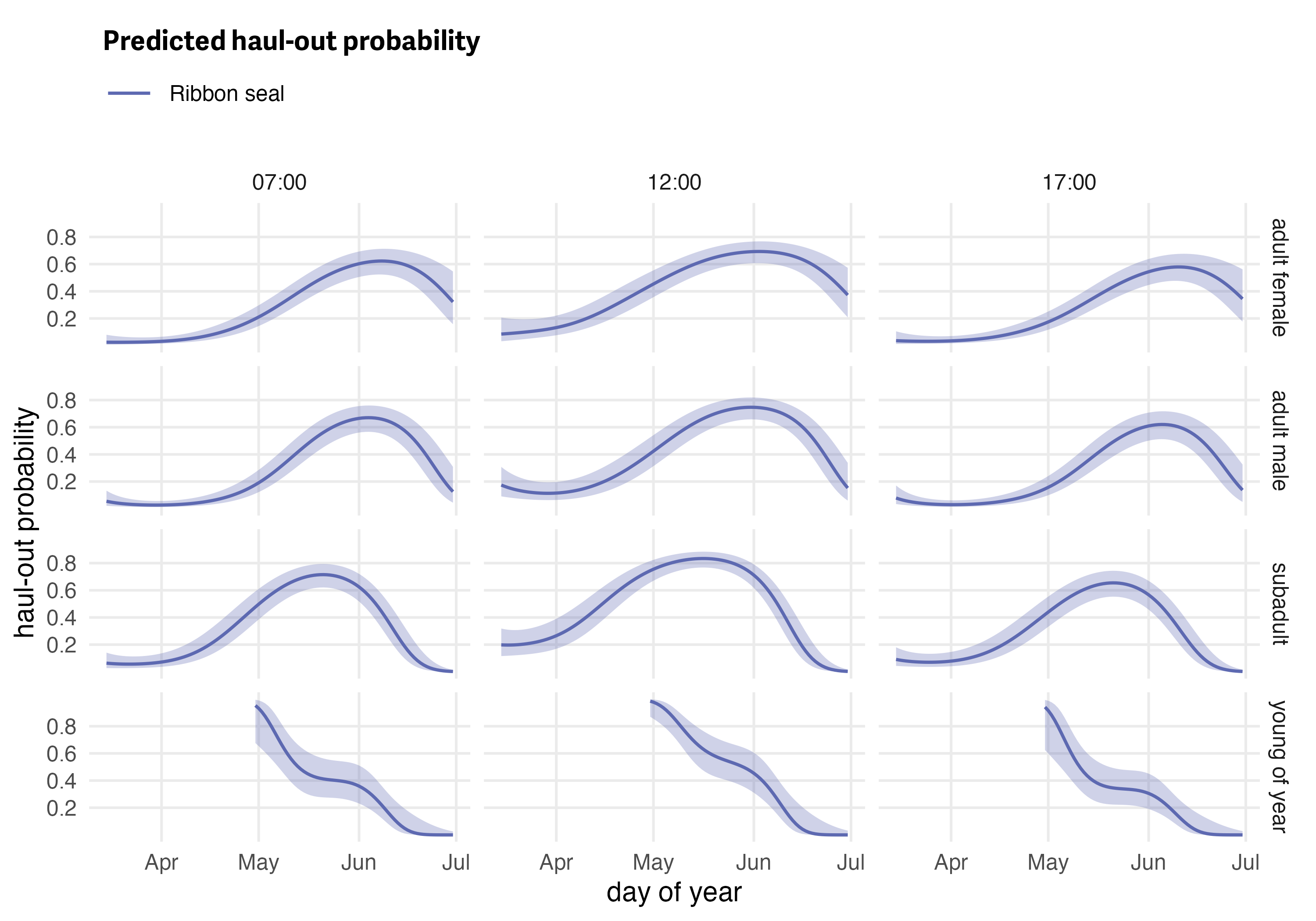

Supplement: Supplemental Information 3 [file peerj-12-18160-s003.png]

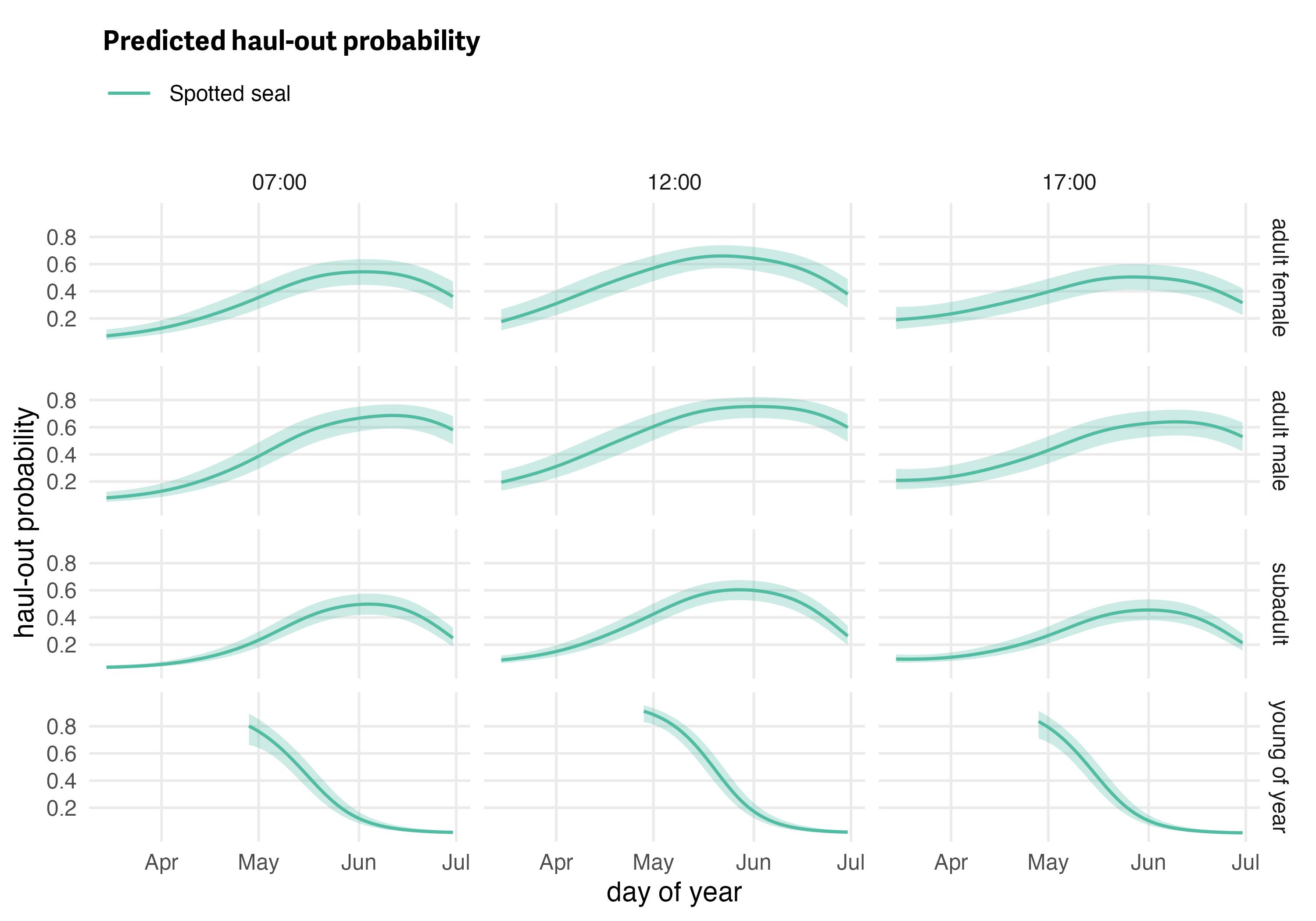

Supplement: Supplemental Information 4 [file peerj-12-18160-s004.png]

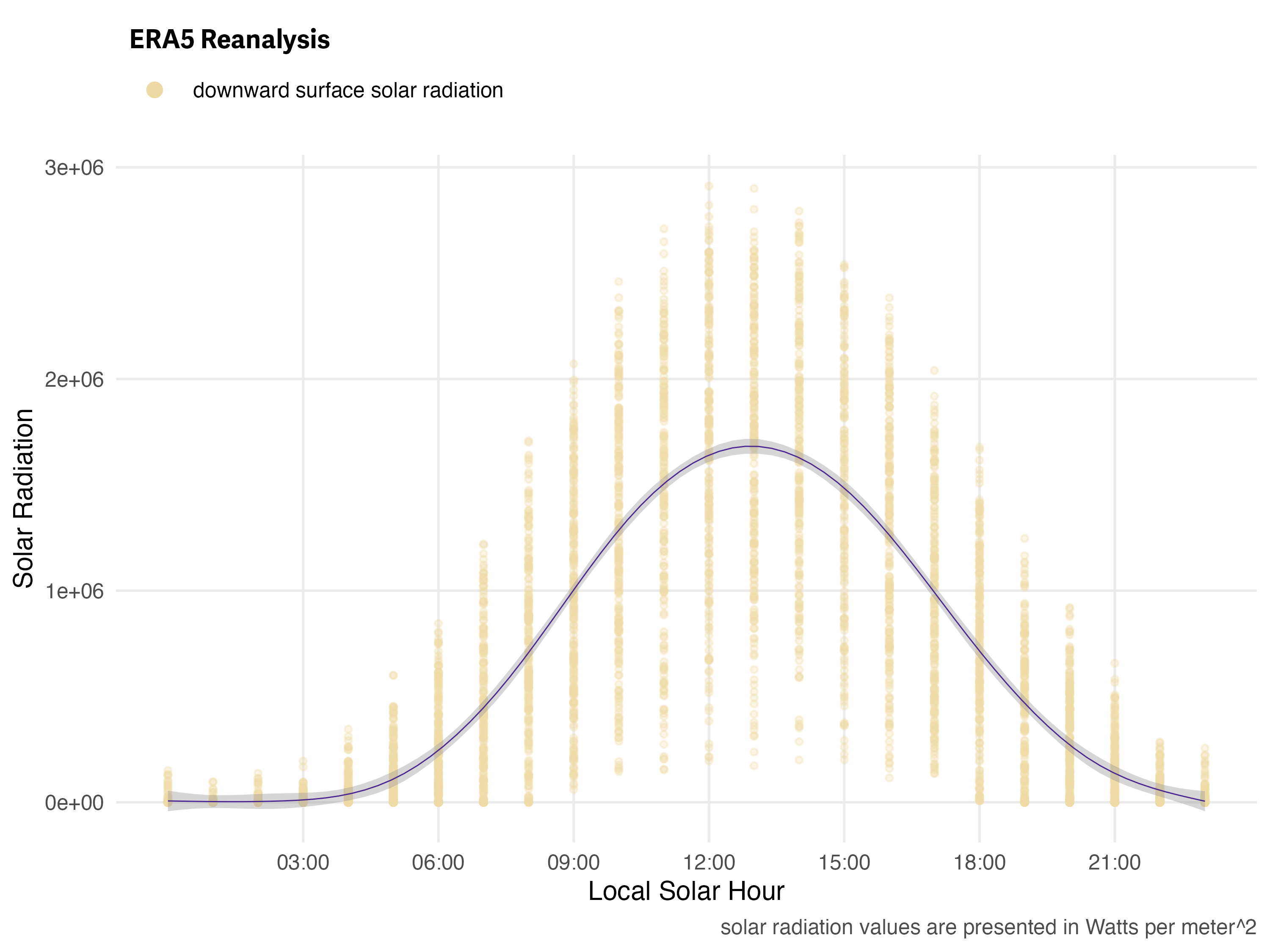

Supplement: Supplemental Information 5 [file peerj-12-18160-s005.png]

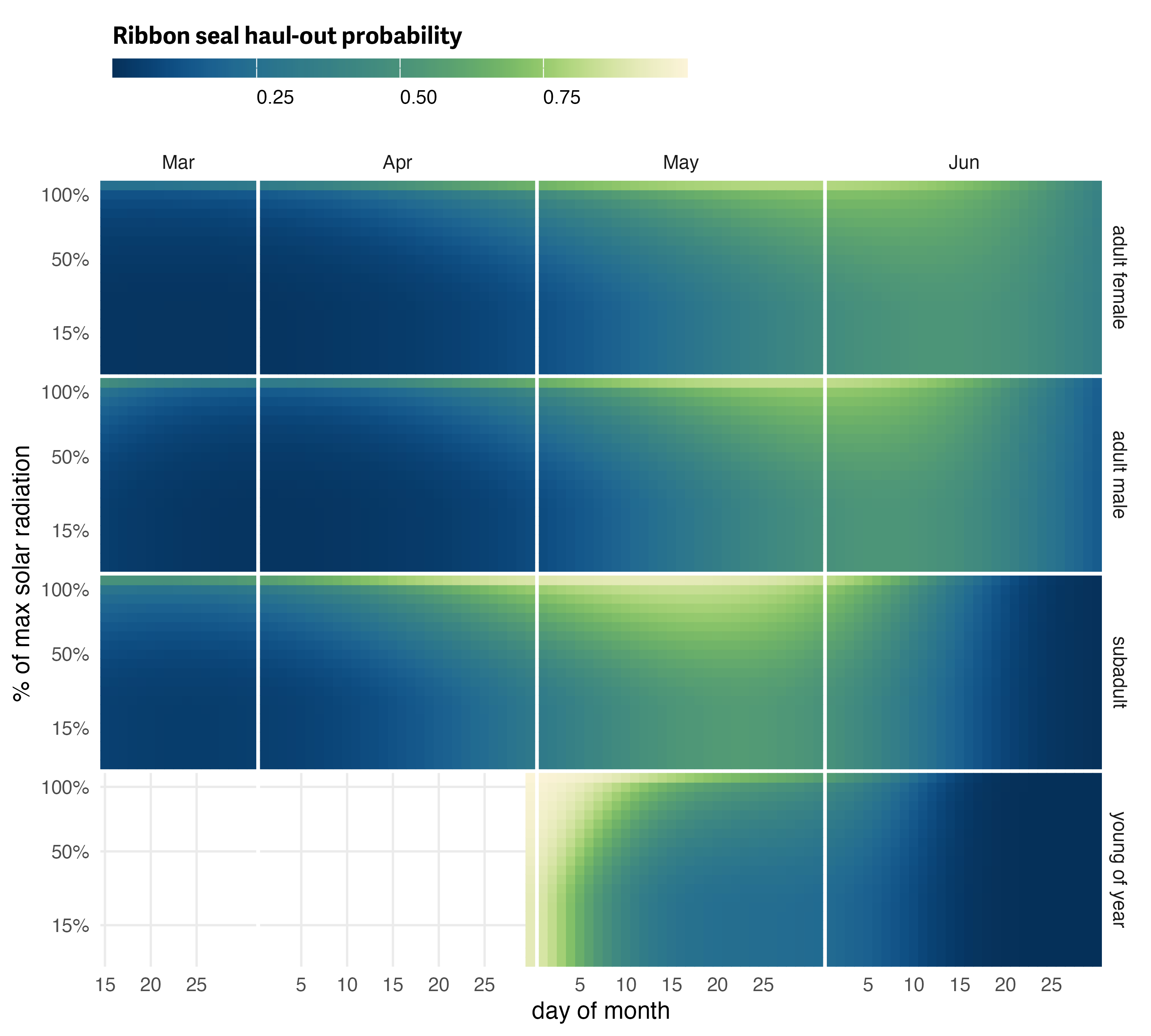

Supplement: Supplemental Information 6 [file peerj-12-18160-s006.png]

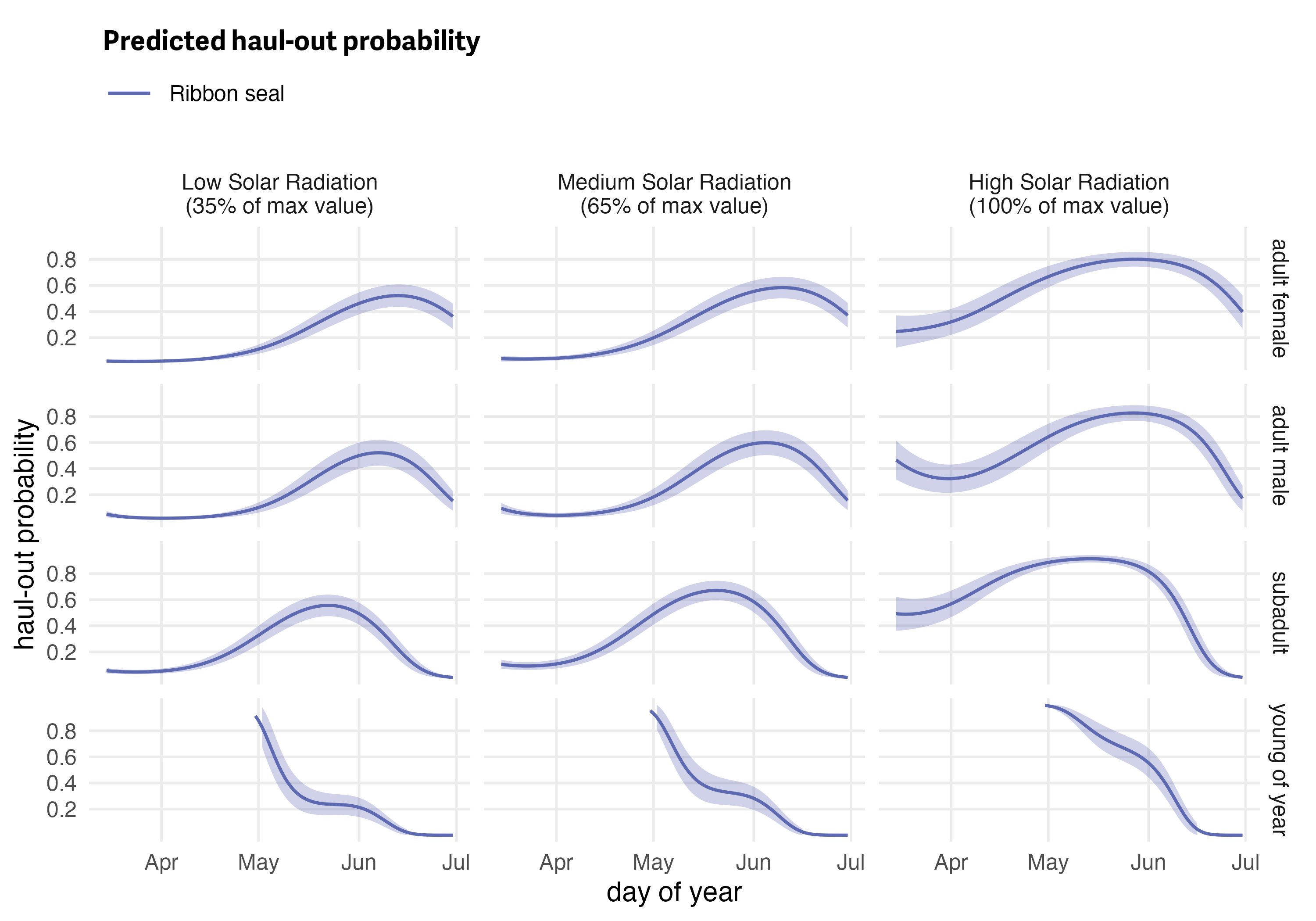

Supplement: Supplemental Information 7 [file peerj-12-18160-s007.png]
